# Design of a New “U”‐shaped Staple and Its Clinical Application in Postoperative Ankle Valgus of Congenital Pseudarthrosis of the Tibia in Children

**DOI:** 10.1111/os.13381

**Published:** 2022-07-20

**Authors:** Xiongke Hu, Anping Li, Kun Liu, Jiangyan Wu, Haibo Mei

**Affiliations:** ^1^ Department of Pediatric Orthopedics, Hunan Children's Hospital Pediatric Academy of University of South China Changsha Hunan China

**Keywords:** Ankle valgus, Congenital pseudarthrosis of the tibia, Guided growth, Hemiepiphyseal block, “U”‐shaped staple

## Abstract

**Objective:**

There has been a lack of suitable epiphysis blocking materials due to the characteristics of less tissue coverage and narrow epiphysis in children's distal tibial medial malleolus. Therefore, this study is to investigate the clinical efficacy and safety of a new “U”‐shaped staple in the treatment of postoperative ankle valgus of congenital pseudarthrosis of the tibia (CPT).

**Method:**

According to the inclusion and exclusion criteria, 33 patients with postoperative ankle valgus of CPT were treated with new “U”‐shaped staples from May 2013 to September 2019. The deformity of ankle valgus was gradually corrected by implanting a new “U”‐shaped staple on the medial side of the distal tibia. Clinical indexes such as the operation time, intraoperative bleeding and hospital stay were observed. Tibiotalar angle was selected as the evaluation index of ankle valgus. American Orthopedic Foot & Ankle Society (AOFAS) scale was used for clinical evaluation of ankle function. The tibiotalar angle, deformity correction rate and complications were evaluated by preoperative, postoperative and last follow‐up imaging data. Student's *t*‐test was used for statistical analysis.

**Results:**

Thirty‐three patients, including 12 males and 21 females were included. All the patients were followed up for at least 14 months, with an average of 35 months. The average operation time was 23 (15–40) min, the average amount of intraoperative bleeding was 7.5 (4–10) mL, and the average hospital stay was 4.2 (3–6) days. The intraoperative tibiotalar angles of all patients were 74.2° ± 4.6°, the tibiotalar angle were 86.8° ± 4.9° when internal fixation was removed, and the tibiotalar angles at the last follow‐up were 84.3° ± 5.9°. The average orthopedic rate was 0.68° per month. No patients suffered from serious complications such as screw prolapse, osteomyelitis, wound infection, etc. Postoperative wound pain complications occurred in two patients, which were relieved after conservative treatment. The AOFAS score improved from 46.2 ± 9.4 before the operation to 74.6 ± 5.7 at the last follow‐up (*P* < 0.01). The ankle movement was good without joint stiffness. There was no epiphyseal plate injury after the removal of internal fixation.

**Conclusion:**

The new “U”‐shaped staple is characterized by simple implantation, low notch, lower risk of fixation failure and close fitting with cortical bone. It is a safe and effective internal fixation system for the treatment of ankle valgus in children.

## Graphical abstract

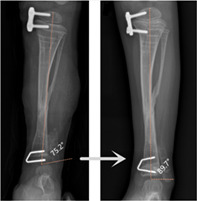

There has been a lack of suitable epiphysis blocking materials due to the characteristics of less tissue coverage and narrow epiphysis in children's distal tibial medial malleolus. The new “U”‐shaped nail designed by us is characterized by simple implantation, low notch, anti‐withdrawal and close fitting with cortical bone. It is a safe and effective internal fixation system for the treatment of ankle valgus in children.

## Introduction

Congenital pseudarthrosis of the tibia (CPT) is a challenging pediatric condition characterized by segmental dysplasia of the tibia, usually accompanied by angular deformity, refractory fracture and nonunion of the affected tibia.^1^, ^2^ Recently, we concluded that the fracture fusion rate can be improved significantly and the recurrence rate of fractures can be reduced apparently by the comprehensive surgical treatment, such as removal of tibial periosteum and pseudo joint, intramedullary staple fixation of foot and ankle, encapsulated autogenous iliac bone graft and Ilizarov external fixation, etc.^3^, ^4^, ^5^, ^6^ However, complications such as unequal length of both lower extremities, ankle valgus and knee valgus are prone to occur after CPT in children.^7^, ^8^ Thabet *et al*.^9^ conducted an average follow‐up of 4.3 years, and found that the incidence of postoperative ankle valgus of CPT was as high as 35%. We treated a total of 231 patients with CPT by combined surgery from August 2008 to September 2018, including 58 patients with postoperative ankle valgus, the incidence was 25.1%. Due to ankle valgus, children will suffer from difficulty in shoe wearing, walking instability or mechanical pain, etc. Ankle valgus also increases the risk of refracture in CPT cases. Generally, conservative treatment (such as analgesic drugs, dynamic and static orthoses etc.) will come with poor effect, therefore, surgical intervention is needed for the treatment of ankle valgus in children.^10^, ^11^

In the 1940s, Phemister put forward firstly the concept of treating children's bone deformities by epiphyseal block.^12^ Subsequently, Stevens further proposed the concept of guided growth based on Phemister's idea.^13^ Combined with engineering mechanics, the growth of one epiphyseal plate was inhibited by internal fixation, and the normal growth of the opposite epiphyseal plate was retained, so as to correct bone angulation deformity. In recent years, a large number of scholars have reported that guided growth technology based on epiphyseal block has achieved satisfactory results in the treatment of knee valgus, ankle valgus and unequal length of both lower extremities in children.^11^, ^14^, ^15^ Compared with osteotomy, hemiepiphyseal fixation has the advantages of simple operation, predictability and low complications, so that it has gradually become a popular choice for children's orthopedists.^16^ Intramedullary rods or Ilizarov external fixation can be used to improve ankle function, but hemiepiphyseal block is the most favorable method to correct ankle valgus in children with CPT.^17^ The guided growth of distal tibia can not only improve ankle valgus, but also reduce the risk of fracture in patients with CPT.^18^ Ankle valgus in children often need to be blocked at the medial malleolus of the distal tibia. However, due to its characteristics of less tissue coverage and narrow epiphysis, it is often difficult to choose the blocking material for internal fixation. Therefore, based on the anatomical characteristics of the distal tibia, we designed a new anatomical “U”‐shaped staple suitable for the included angle between the medial cortical bone of the distal tibia and the epiphyseal horizontal line, which has the characteristics of low notch, lower risk of fixation failure and close fitting to the tibial cortex.

In this retrospective study, our preliminary study included the following: (i) to evaluate the clinical efficacy and safety of a new “U”‐shaped staple in the treatment of postoperative ankle valgus of CPT; (ii) compared with the hemiepiphyseal block in the treatment of ankle valgus reported by other scholars, does the new “U”‐shaped staple have advantages? and (iii) to clarify the working principle of the new “U”‐shaped staple and provide a new choice of internal fixation for the hemiepiphyseal block treatment of ankle valgus in children.

## Patients and Methods

### Study Population

*Inclusion criteria*: (i) patients with CPT developed ankle valgus after surgery, and they were performed with epiphyseal block treatment with “U”‐shaped staple; (ii) patients 2–14 years old, and had immature skeleton; (iii) the existence of ankle valgus deformity was indicated by the X‐ray film of both lower extremities in standing position; and (iv) the tibiotalar angle was used as an observation index of ankle valgus.

Exclusion criteria: (i) ankle valgus deformity that was caused by traumatic epiphyseal plate premature closure, epiphyseal plate lesion after infection and other reasons; (ii) patients with CPT who developed ankle valgus after surgery, and were treated with epiphyseal block treatment with other internal fixation materials; and (iii) patients who missed follow‐up.

### Patient Population

In line with the criteria abovementioned, we included a total of 33 patients into the study, including 12 males and 21 females. This study was approved by the Ethic Committee (KYSQ2021‐077) and the consent of the guardian.

### “U”‐shaped Staples

**Fig. 1:**
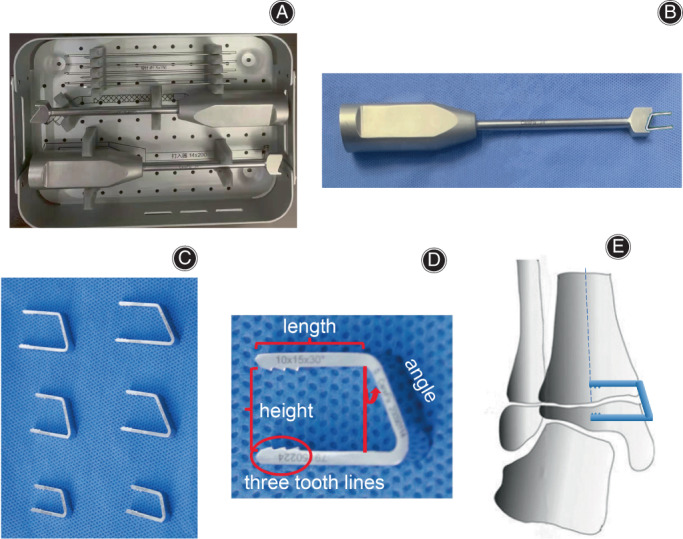
Introduction of new “U”‐shaped staples. (A) Tool box for new “U”‐shaped staples; (B) Supporting tools can be used for clamping “U”‐shaped staples well to ensure stability and facilitate impact implantation; (C) The new “U”‐shaped staple has six specifications; (D) The detailed drawing of 10 × 15 × 30°”U″‐shaped staples. The first value represents height, the second value represents length and the third value represents angle. In addition, three tooth lines are designed on the screw head, which can effectively reduce the risk of screw withdrawal; (E) A schematic figure of “U”‐ shaped staple implantation.

The “U”‐shaped staples are stainless steel forged according to Chinese GB 4234.1 standard. The design of “U”‐shaped staples refers to yyt0956‐2014 standard and is applied in clinic in accordance with the approval of *Administrative Measures for Registration and Filing of Medical Devices in China*. The thickness of the new “U”‐shaped staple is 1.5 mm, and the width of the screw and three tooth lines on the head are added. We measured the angle between the medial malleolus of the distal tibia and epiphysis, and designed two specifications of anatomical “U”‐shaped staples of 24° and 30° (Fig. 1). The new “U”‐shaped staple independently developed by us, equipped with special implantation tools, is characterized by simple operation, low notch, anti‐withdrawal and close fitting with cortical bone.

### Surgical Procedure

#### Anesthesia and Exposure

The patients were administrated with antibiotics for preoperative prevention, and were administered general anesthesia in the supine position. We determined the position of medial epiphyseal plate of distal tibia by C‐arm fluoroscopy.

*Surgical Exposure and “U”‐shaped Staples Implantation*. We made a skin incision on the medial side of the distal tibia, with the length of about 3 cm, exposing the periosteum layer by layer, so as to avoid injury of blood vessels and nerves. Before the operation, the distance between epiphyseal plate and epiphyseal midline was measured by means of imaging, and “U”‐shaped staples with appropriate height were selected to ensure that epiphyseal plate was at the midline level of fixation by “U”‐shaped staples. The distance between the distal tibial cortex and the central axis was measured, and “U”‐shaped staple with the appropriate length was selected, 24° or 30° U‐shaped staples were selected according to the included angle between the medial cortical bone of the distal tibia and the epiphyseal horizontal line. The “U”‐shaped staple was implanted in parallel to the epiphyseal plate line through a special auxiliary implantation tool. The position of the implant was checked by using anteroposterior and lateral X‐ray images obtained by intraoperative fluoroscopy.

#### Closing

We rinsed the wound, deflated the tourniquet, compressed wound and stopped bleeding by electrocautery. We placed the anti‐adhesion film on the wound to prevent postoperative scar adhesion, and used the absorbable suture to suture the wound.

#### Clinical Outcomes

Clinical outcomes were assessed by an experienced clinical research coordinator. Functional data include The American Orthopedic Foot & Ankle Society (AOFAS) ankle‐hindfoot scale. These data were collected before surgery, at 3 months, after removal of internal fixation, and the last follow‐up.

##### Duration of Surgery

The duration of surgery was defined as the time to perform all procedures after anesthesia, including positioning the patient, exposing the surgical area, “U”‐shaped Staples Implantation, wound suture.

##### Intraoperative Blood Loss

Intraoperative blood loss refers to the blood loss during the operation, which is measured by the total amount of blood in the suction bottle and gauze.

##### Length of Hospitalization

Length of hospitalization was defined as the number of days the patient stayed in the hospital, which was calculated from the date of admission to the date of discharge.

##### AOFAS Scale

The AOFAS scale was used to evaluate foot and ankle function. The AOFAS scale includes nine sections: pain intensity; functions, autonomous activities and support situation; maximum walking distance; ground walking; abnormal gait; front and rear activities (buckling and stretching); hindfoot activities (inversion and eversion); ankle ‐ hindfoot stability (anteroposterior, inversion and eversion); foot alignment. Each section comprises two or four statements that are scored from 0 to 40. Here, less than 50 points is considered bad, 50–74 points is considered fair, 75–89 points is considered medium, and 90–100 points is considered good.

### Radiographic Evaluation

After the operation, the patients were performed with reexamination every 3–4 months, and conducted with the X‐ray films of both lower extremities standing to monitor the limb growth and deformity correction. We recorded the tibiotalar angle every time. When the ankle valgus deformity is corrected, the internal fixation should be taken out.

#### Tibiotalar Angle

**Fig. 2:**
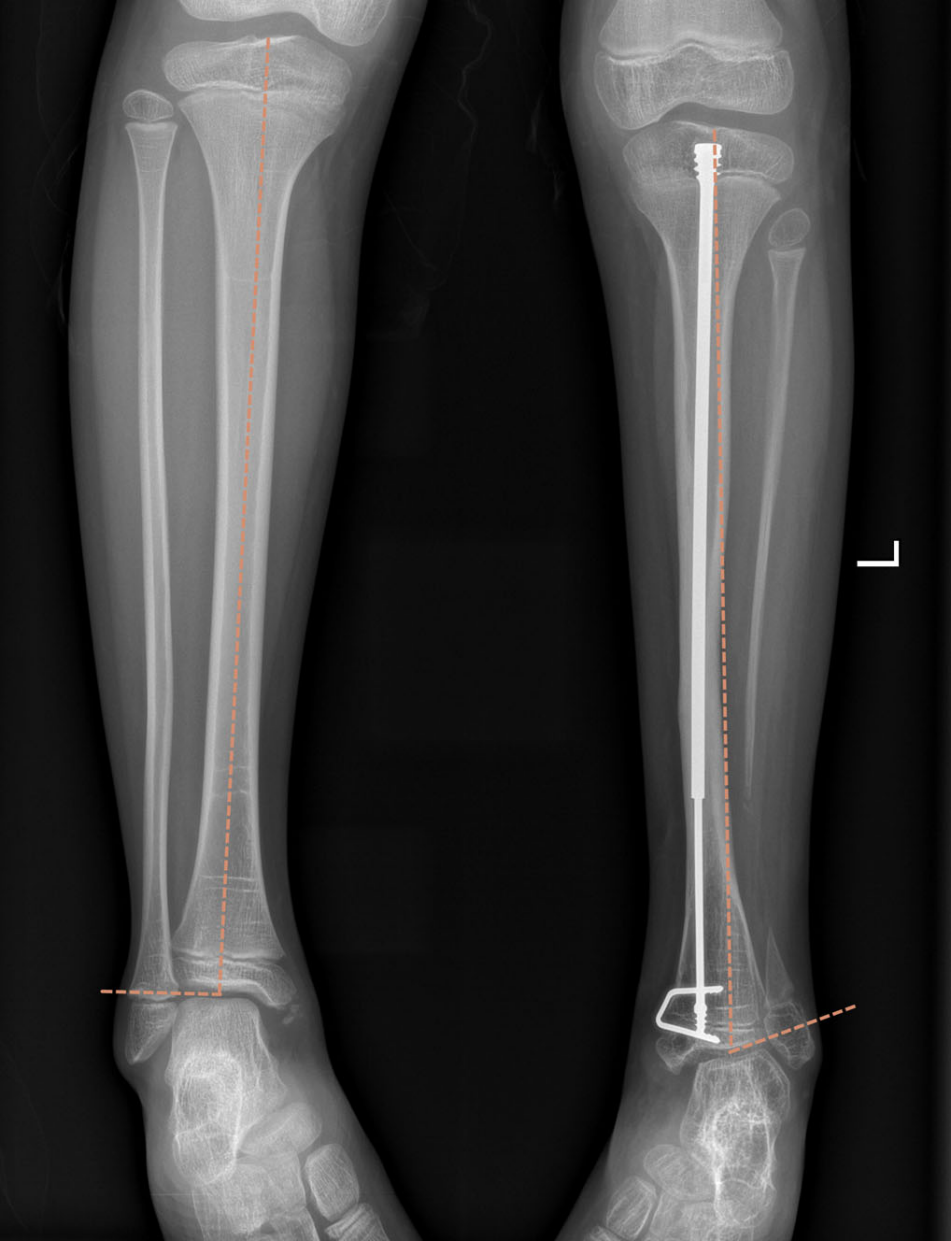
As for the measurement of tibiotalar angle in children with CPT, the included angle between the midpoint line between the center of tibial intercondylar ridge and the level of ankle space and the articular surface of talus fornix was selected.

Due to tibia bending which often occurs in the patients with CPT, the included angle between the articular surface of talar fornix and the midpoint line crossing the center of tibial intercondylar ridge and the level of ankle joint space was chosen (Fig. 2). The normal range of tibiotalar angle is 80–90°, and valgus within 10°can be corrected as much as possible through the movement of hindfoot joint.^19^

## Results

### General Results and Clinical Outcomes

There were 33 patients, including 12 males and 21 females. All the patients were had follow up visits for at least 14 months, with an average of 35 months. The average operation time was 23 minutes, the average amount of intraoperative bleeding was 7.5 mL, and the average hospital stay was 4.2 days. The AOFAS score improved from 46.2 ± 9.4 before the operation to 74.6 ± 5.7 at the last follow‐up (*P* < 0.01) (Table 1).

**TABLE 1:** Patients' Characteristics

	Count
Ankle (n)	33
Age at initial surgery (y)	6.9 ± 3.4
Age at hardware removal (y)	8.4 ± 3.2
Sex (n)	
Male	12
Female	21
Tibiotalar angle (°)	
At initial surgery	74.2 ± 4.6
At hardware removal	86.8 ± 4.9
At the last follow‐up	84.3 ± 5.9*
American Orthopedic Foot & Ankle Society scale	
At initial surgery	46.2 ± 9.4
At the last follow‐up	74.6 ± 5.7*

* Indicates a statistically significant difference between preoperative and the last follow‐up of tibiotalar angle (*P* = 0.02); ^#^Indicates a statistically significant difference between preoperative and the last follow‐up of AOFAS scale (*P* = 0.00).

### Radiographic Results

**Fig. 3:**
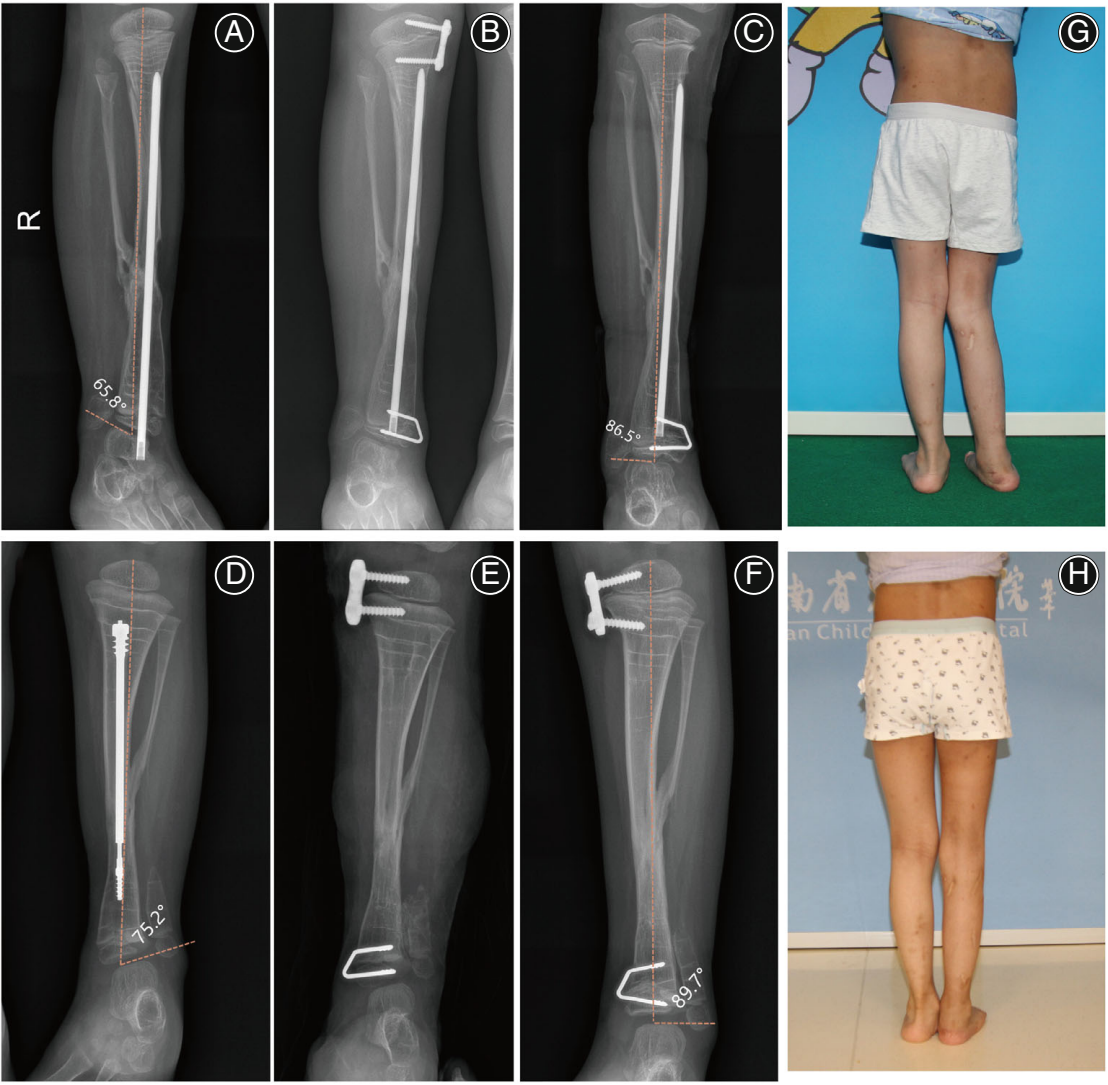
**(**A) Male, 2 years and 3 months old, underwent “tibial pseudoarthrectomy + intramedullary staple fixation + encapsulated bone graft + Ilizarov external fixation” due to CPT. The affected ankle valgus deformity occurred 3 years after operation; (B) Postoperative X‐ray showed that the tension band screw was in good position and fitted with the medial tibial cortex; (C) After 10 months of guided growth, the correction of ankle valgus deformity was satisfactory; (D) Male, 1 year and 8 months old, underwent “tibial pseudoarthrectomy + extensible intramedullary staple fixation + encapsulated bone graft + Ilizarov external fixation” due to CPT. Severe ankle valgus deformity occurred on the affected side 3 years and 2 months after operation; (E) Postoperative film showed that the tension band screw was in good position; (F) The ankle valgus deformity was completely corrected 15 months after operation; (G and H) This is the clinical gross photograph of the patient in Fig. 4A‐C. It can be seen that the appearance of the patient's right ankle valgus has improved significantly.

**Fig. 4:**
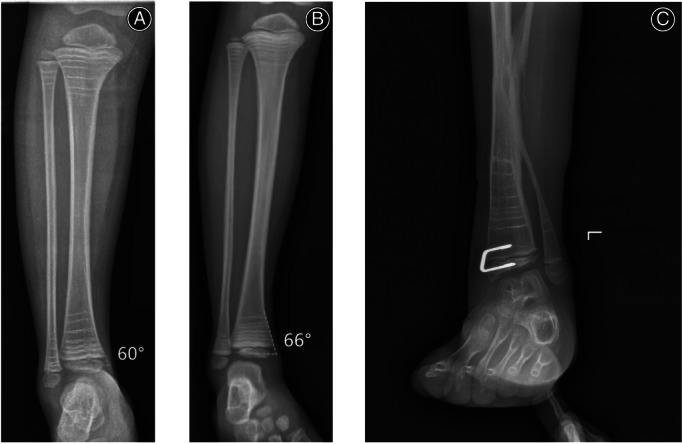
(A) The included angle between the epiphyseal axis of the distal tibia and the cortical bone of a 4‐year‐old normal child is 60°; (B) The included angle between the epiphyseal axis of distal tibia and cortical bone in a 6‐year‐old normal child is 66°. We measured the cortical bone angle of the distal tibia of 80 normal children aged 2–12 years, and the result was 65.1° ± 3.2°. (C) A 6‐year‐old girl with postoperative ankle valgus of CPT was treated with 10° × 15° × 24° “U”‐shaped staple, which could fit the bone cortex well, and the orthopedic effect was enhanced, screw loosening was avoided as well.

The intraoperative tibiotalar angles of all the patients were 74.2° ± 4.6°, the tibiotalar angle were 86.8° ± 4.9° when internal fixation was removed, and the tibiotalar angles at the last follow‐up (84.3° ± 5.9°) (Figs 3 and 4). There was a significant difference of the tibial‐talar angle in the last follow‐up and preoperative (*P* = 0.02). The average orthopedic rate was 0.68° per month.

### Statistical Analysis

All the statistics were calculated using SPSS version 20.0 (Chicago, IL, USA). Continuous variables including age, duration of surgery, intraoperative blood loss, length of hospitalization are reported as mean ± standard deviation. Statistical analysis was conducted by using the student's *t* ‐test to assess statistical differences, *P* < 0.05 was considered statistically significant.

## Discussion

It is generally considered that the guided growth technique of epiphyseal plate block is an effective method for the treatment of limb deformity and unequal length of both lower extremities in children. Due to less tissue coverage and narrow epiphysis in children's distal tibial medial malleolus, and the requirements for epiphysis blocking materials are relatively high. The new “U”‐shaped staple designed by us has the characteristics of low notch, lower risk of fixation failure and close fitting with cortical bone. It has already been successfully applied in postoperative ankle valgus of CPT in children.

### Postoperative Ankle Valgus of CPT

Because of segmental skeletal dysplasia, CPT is a troublesome disease in pediatric orthopedics, often accompanied by nonunion and tibial deformity. Presently, the commonly used surgical treatment methods mainly include intramedullary staple fixation, Ilizarov external fixation, combination of intramedullary staple and Ilizarov external fixation, as well as vascularized fibula transplantation.^4^, ^5^, ^20^ Agashe *et al*.^21^ treated 15 patients with CPT by using tibial intramedullary staple and Ilizarov external fixation, of which 14 cases achieved bone healing, but seven cases had ankle valgus deformity. A total of 56 patients with CPT were treated with tibial intramedullary staple combined with encapsulated bone graft and Ilizarov external fixation. After an average of 8.5 years of long‐term follow‐up, it was found that the healing rate of postoperative fracture reached 89.2%, but the incidence of ankle valgus deformity was 17.9%.^4^ Patients with CPT might easily suffer from the proximal migration of distal fibula and ankle valgus deformity after ankle intramedullary nailing. So, patients with CPT are recommended to be closely monitored in the ankle function in long‐term treatment.

### Hemiepiphyseal Block for Ankle Valgus

The surgical treatment of ankle valgus in children mainly includes supra‐ankle osteotomy and hemiepiphyseal block.^10^, ^11^ It has been proved that hemiepiphyseal block‐based guided growth technology is an effective treatment for the correction of skeletal malformations in immature children. Compared with supra‐ankle osteotomy, hemiepiphyseal block has the advantages of less trauma, less complications and rapid postoperative recovery.^16^, ^22^ At present, hemiepiphyseal block commonly used in distal tibia includes tension band screws and trans‐epiphyseal plate screws. Because the trans‐epiphyseal plate screw passes through the epiphyseal plate, it is easy to come with complications such as epiphyseal plate bone bridge and difficulty of removing the screw growing into the bone cortex.^23^, ^24^ At present, the clinical application of trans‐epiphyseal plate screw in distal tibia is gradually reduced. Previously, tension screws commonly used for hemiepiphyseal block include “8”‐shaped plate screws and Blonut staples. The “8”‐shaped plate screw has the advantage of flexible screw angle, but the thickness of the steel plate and the screw tail often leads to too high internal fixation notch. The medial soft tissue of ankle joint is less and there is a lack of muscle tissue coverage. The internal fixation of high notch is easy to stimulate the skin, resulting in complications of pain and skin ulceration.

### Characteristics of New “U”‐shaped Staple

**Fig. 5:**
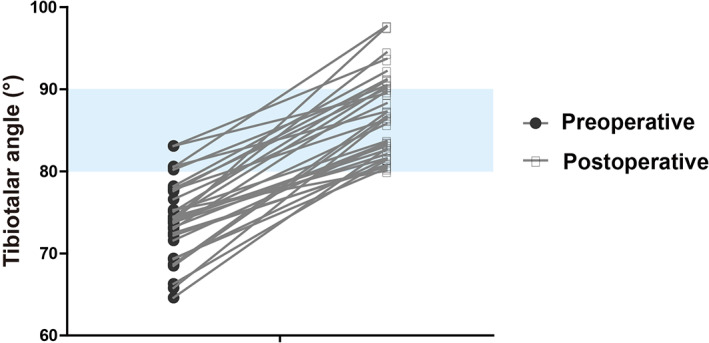
The Corresponding Changes of Tibiotalar Angle in Each Patient

The thickness of the new “U”‐shaped staple designed by us is 1.5 mm, and the notch after implantation is lower than that of the “8”‐shaped plate screw. Blount staples are inserted into the epiphysis of children who are not completely ossified. Staples can withdraw with growth.^25^, ^26^ The new “U”‐shaped staple is similar to the improved version of Blount stapler. It adds the width of the screw and three tooth lines on the head. Compared with the tension screw without tooth lines, it effectively reduces the risk of screw withdrawal. According to the literature, the probability of internal fixation loosening and migration of Blount stapler is about 9.7%–14.6%.^27^, ^28^ We used the new “U”‐shaped staple to treat 33 children without internal fixation loosening, which indicates that the risk of internal fixation failure of the “U”‐shaped staple is relatively low. In addition, based on the anatomical characteristics of the medial malleolus of the distal tibia, a special angle was introduced, temporarily named “the included angle between the medial cortical bone of the distal tibia and the epiphysis,” that is the included angle between the epiphysis axis of the distal tibia and the medial cortical bone. We measured the angle between the medial malleolus of the distal tibia and epiphysis in 80 normal children aged 2–12 years, and the result was 65.1° ± 3.2° (Fig. 5). Therefore, we creatively designed two specifications of anatomical “U”‐shaped staples of 24° and 30° (Fig. 5). When the cortical bone angle of the distal tibia was close to 60°, the 30° “U”‐shaped staple was selected, and when the cortical bone angle of the distal tibia was close to 66°, the 24° “U”‐shaped staple was selected. In all 33 patients, 15 patients used 30° “U”‐shaped staples and 18 patients used 24° “U”‐shaped staples. Based on the design of this angle, the “U”‐shaped staple can be closer to the bone cortex, so as to enhance the holding force of the screw, ensure better blocking effect, and the screw is not easy to loosen and prolapse. Presently, we have temporarily made six types of “U”‐shaped staples, with two heights of 10 and 15 mm, two lengths of 15 and 20 mm and two angles of 24° and 30°, which can basically meet the needs of distal tibial epiphyseal plate block in children of different ages. The “U”‐shaped staples are designed based on the included angle between the medial cortical bone of the distal tibia and the epiphysis, and they are mainly derived from the measurement of a large number of children's X‐ray data. Subsequent anatomical evidence and prospective studies need to be supplemented to further illustrate the reliability of this angle. The new “U”‐shaped staple, equipped with special implantation tools, is characterized by simple operation, low notch, anti‐withdrawal and close fitting with cortical bone.

### Therapeutic Outcome

We treated 33 children with postoperative ankle valgus of CPT with new “U”‐shaped staples. The results showed that the tibiotalar angle was corrected from preoperative 74.2 ± 4.6° to postoperative 86.8 ± 4.9°. The average orthopedic rate was 0.68° per month. Driscoll *et al*.^29^ reported that 25 children with ankle valgus were treated with tension band screws, with an average age of 10.9 years. The postoperative results showed that the average correction rate of tension band screws was 0.36° per month. Rupprecht *et al*.^30^ treated nine children with hereditary multiple exostosis with ankle valgus deformity by using a distal tibial medial epiphyseal plate screw. The average age was 11.8 years at the time of operation and the average monthly correction rate was 0.58°. It is found that the correction of ankle valgus is significantly associated with the age of patients, and better orthopedic improvement often occurs for young patients.^11^, ^13^, ^31^ In our study, due to the early onset of CPT and occurrence of ankle valgus deformity in the young patients after surgical operation, the orthopedic effect is relatively better than the that reported by other scholars. For example, the average age of our new “U”‐shaped staple group was 6.9 years old. Because the age and growth rate of different patients are not completely consistent, it is difficult to accurately predict the individual correction rate. Therefore, children receiving distal tibial hemiepiphyseal block need close imaging follow‐up until bone maturity. None of the patients treated with new “U”‐shaped staples suffered from serious complications such as screw prolapse, osteomyelitis, and wound infection. The AOFAS score improved from 46.2 ± 9.4 before the operation to 74.6 ± 5.7 at the last follow‐up (*P* < 0.05). They had good ankle movement and no joint stiffness. There was no epiphyseal plate injury after the removal of internal fixation.

### Limitations

There are also many limitations in this article. First, because it is a retrospective study, the collection of preoperative clinical and imaging data is lack of unity, and there is a certain selection deviation. Second, the influence of patients' age or gender on orthopedic effect has not been discussed, and more strict grouping can be considered subsequently. Finally, due to the follow‐up time of this study being short, longer‐term follow‐up and prospective research should be carried out.

## Conclusion

In general, temporary epiphyseal plate block by implanting new “U”‐shaped staples in the medial distal tibia is a safe and effective method for the treatment of postoperative ankle valgus of CPT in children. The new “U”‐shaped staple is characterized by simple implantation, low notch, lower risk of fixation failure and close fitting with cortical bone. It is a good choice of implant materials for distal tibial epiphyseal plate block. Of course, in the later stage, we will develop more models and angles of “U”‐shaped staples, and conduct the more accurate treatment based on the imaging examination of patients.

## Ethics Approval and Consent to Participate

Ethical approval was obtained from the Hunan Children's Hospital Ethics Committee. Written informed consent was obtained from individual or guardian participants.

## Authors Contributions

Xiongke Hu and Haibo Mei carried out the majority of the study, analyzed data and prepared the manuscript. Xiongke Hu and Anping Li supervised the study and wrote the first draft of manuscript. Jiangyan Wu and Kun Liu assisted with the study and the analysis of the data. All authors approved the final manuscript.

## Funding

This work was supported by the Natural Science Foundation of Hunan Province, China (Grant No. 2020JJ5900), the Clinical Research Center for Limb Deformity of Children in Hunan Province(Grant No. 2019SK4006), the Project of Hunan Provincial Health Committee (Grant No. 202204083923), the Project of the Administration of Traditional Chinese Medicine of Hunan Province (Grant No. D2022083), the Clinical Research (Translation) Center of Hunan Children's Hospital.
